# P01-05 Development of the MOVING policy framework: monitoring and promoting action in physical activity policy

**DOI:** 10.1093/eurpub/ckac095.005

**Published:** 2022-08-29

**Authors:** Kate Oldridge-Turner, Margarita Kokkorou, Fiona Sing, Ioana Vlad, Diva Fanian, Arnfinn Helleve, Knut-Inge Klepp, Harry Rutter, Kate Allen

**Affiliations:** 1 World Cancer Research Fund International, London, United Kingdom; 2 Norwegian Institute for Public Health, Oslo, Norway; 3 Department of Social and Policy Sciences, University of Bath, Bath, United Kingdom

**Keywords:** Physical activity policy, policy database, policy actions, policy framework

## Abstract

**Background:**

The World Cancer Research Fund International's (WCRF International) analysis of global research shows a strong link between diet, physical activity, and risk of cancer. To address this, WCRF International has developed a package of policy resources to promote healthy diets and physical activity to support reporting, categorising and monitoring of policy actions. The MOVING policy framework was developed as a complement to the well-established NOURISHING framework of diet-related policies-outlining a comprehensive set of areas in which governments should take action to promote physical activity. The new framework forms the basis for the MOVING database of implemented policy actions.

**Methods:**

For the MOVING framework, literature reviews were undertaken, and the results were distilled into policy categories on which academics and policy experts provided feedback. The framework is the basis for a specific inclusion criteria and search strategy to determine how policy actions will be collected, reviewed and categorised for the MOVING database. A comprehensive scanning methodology was designed to identify all relevant policies across the MOVING policy areas. Relevant policies are then verified with in-country experts and uploaded to the database. This methodology was tested, refined and will be applied to 27 European countries.

**Results:**

The MOVING policy framework comprises six policy areas within four policy domains and details the areas where governments should take action to promote physical activity. The framework forms the structure for a new database of global physical activity policies, which was launched in the summer of 2020. The MOVING database currently includes 234 policy actions from 12 countries. The MOVING database is a logical and practical tool, allowing users to search for policy actions categorised in the structure of the MOVING framework.

**Conclusion:**

The MOVING physical activity framework and database are innovative tools that support reporting, categorising and monitoring of physical activity policy actions that will work alongside the NOURISHING framework and database. These physical activity policy tools allow stakeholders such as researchers, civil society organizations and policy makers to quickly identify both gaps and strengths in government action and therefore assess where there is scope for improvement within and across countries.

